# Strategies for Outcrossing and Genetic Manipulation of Drosophila Compound Autosome Stocks

**DOI:** 10.1534/g3.112.004481

**Published:** 2013-01-01

**Authors:** Torcato Martins, Shaila Kotadia, Nicolas Malmanche, Claudio E. Sunkel, William Sullivan

**Affiliations:** * Instituto de Biologia Molecular e Celular, Universidade do Porto, 4150-180 Porto, Portugal, and; † Department of Molecular, Cellular and; ‡ ICBAS-Instituto de Ciências Biomédicas de Abel Salazar, and Developmental Biology, University of California, Santa Cruz, Santa Cruz, California 95064

**Keywords:** C(2)EN, long chromosome, HisH2Av-mRFP1, neuroblast, mitosis

## Abstract

Among all organisms, *Drosophila melanogaster* has the most extensive well-characterized collection of large-scale chromosome rearrangements. Compound chromosomes, rearrangements in which homologous chromosome arms share a centromere, have proven especially useful in genetic-based surveys of the entire genome. However, their potential has not been fully realized because compound autosome stocks are refractile to standard genetic manipulations: if outcrossed, they yield inviable aneuploid progeny. Here we describe two strategies, cold-shock and use of the *bubR1* mutant alleles, to produce nullo gametes through nondisjunction. These gametes are complementary to the compound chromosome−bearing gametes and thus produce viable progeny. Using these techniques, we created a compound chromosome two C(2)EN stock bearing a red fluorescent protein-histone transgene, facilitating live analysis of these unusually long chromosomes.

Although much of genetic analysis has focused on the structure and function of individual genes, large-scale chromosome rearrangements also have played an important role in understanding higher levels of genome organization. In fact, the first functional genome-wide screen for regions of haploinsufficiency was achieved using a comprehensive collection of well-defined Y-autosome translocations (Lindsley *et al.* 1972). Chromosome rearrangements also have been essential for defining long-range interactions regulating gene expression and chromatin organization (Girton and Johansen 2008). One class of rearrangements, the compound chromosomes in which both homologs share a common centromere, have proven especially useful in functional genomic studies. For example, compound chromosomes have facilitated genome-wide screens for genes that must be zygotically expressed for completion of embryonic cellularization and gastrulation (Merrill *et al.* 1988; Wieschaus and Sweeton 1988).

Compound chromosomes are generated in a stepwise fashion using a series of complementary translocations, resulting in a doubling of the chromosome arm length (Ashburner 1989; Novitski *et al.* 1981). The arms are linked together with Y-heterochromatin, which cytologically appear as constrictions in the middle of the compounds arms (Figure 1, arrows). Compound chromosomes for the entire second and third chromosomes are referred to as C(2)EN and C(3)EN. For example, C(2)EN consists of both homologs of chromosome 2 sharing a single common centromere, creating a metacentric chromosome with arms twice the normal length (Figure 1). The structure of C(2)EN is 2R-Yhc-2L-C-2L-Yhc-2R. Despite these rearrangements, compound chromosome bearing flies are euploid, viable, and fertile. However, compound chromosome-bearing sperm are selectively lost after insemination (Dernburg *et al.* 1996). Because of their unusually long arm length, the compound chromosomes have been useful in examining the influence of chromosome arm length on chromosome segregation in different cell types. Studies analyzing C(2)EN syncytial embryos revealed that increased arm length resulted in an increased rate of errors in chromosome congression and segregation and loss of the damaged nuclei from the cortex (Sullivan *et al.* 1993). In contrast, a similar analysis in the slower dividing neuroblasts revealed that while the long C(2)EN chromosomes clearly lagged during anaphase, division failures did not occur (Gonzalez *et al.* 1991; Sullivan *et al.* 1993; Kotadia *et al.* 2012). Thus, the rapid maternally driven embryonic divisions were much more sensitive to division errors than were the later zygotic divisions.

**Figure 1:**
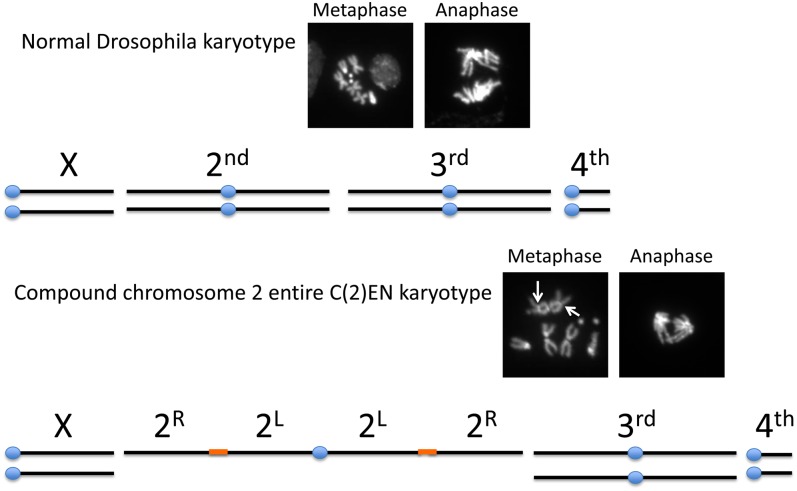
Karyotype of compound chromosome 2, C(2)EN. The normal *Drosophila melanogaster* karyotype contains two distinct X, 2nd, 3rd, and 4th chromosomes. In contrast, C(2)EN flies have two distinct X, 3rd, and 4th chromosomes but both chromosome 2 homologs share a common centromere (blue dot). Construction of the compound chromosome relied on Y-heterochromatin (orange) to physically link left and right arms. DAPI stained images of wild-type and C(2)EN-bearing neuroblasts highlight the constrictions at the Y-heterochromatin (metaphase, arrows) and the unusually long arms of the compound chromosomes (anaphase). The bright spots on the lagging chromosomes in C(2)EN mark the Y-heterochromatin linkers.

A major factor limiting the use of compound chromosomes is the fact that viable progeny are produced only when they are maintained as a stock. For example, the C(2)EN stock produces three progeny classes bearing no, four or two copies of chromosome 2. The first two classes are aneuploid and only the latter class produces viable fertile progeny (Figure 2A). If the C(2)EN stock is outcrossed, only inviable aneuploid progeny containing either one or three copies of chromosome 2 are produced (Figure 2B). Consequently, these stocks have been refractile to traditional genetic analysis such as introducing mutant alleles and transgenes into the stock.

**Figure 2:**
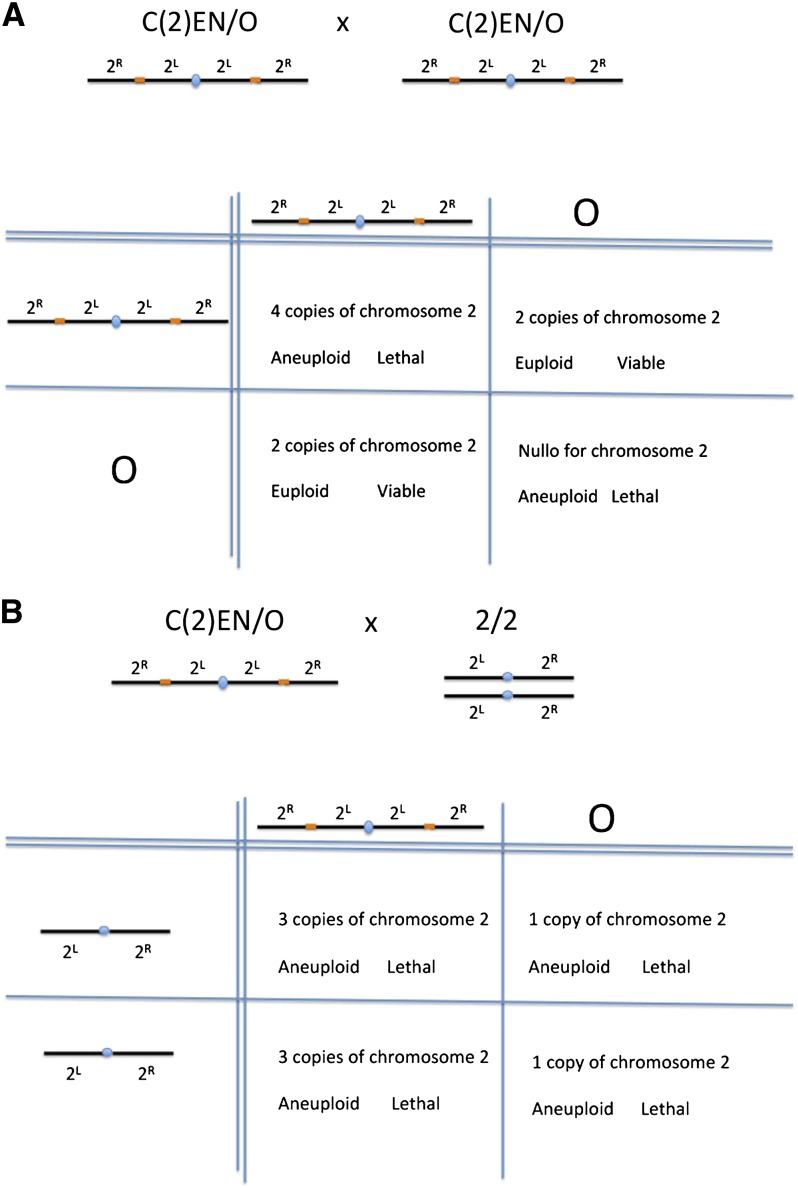
C(2)EN is maintained as a stock but cannot be outcrossed. (A) Crossing C(2)EN-bearing males and females results in 50% inviable progeny containing either two or no copies of the compound chromosome. The remaining 50% are euploid and viable containing a single copy of the compound chromosome. (B) All of the progeny derived from outcrossing C(2)EN-bearing individuals to wild-type *Drosophila melanogaster* are aneuploid and inviable, containing either one or three copies of the second chromosome.

Here we describe two strategies for producing viable progeny from outcrossed compound stocks. Both strategies are based on increased nondisjunction rates such that mutant alleles and transgenes can be stably introduced into the compound chromosome stocks. In our first protocol, we induce high rates of nondisjunction through cold-shock as described by Ashburner (1989). Typically, ~300 virgin females bearing a second chromosome balancer with a dominant marker, for example CyO, are collected. Virgin female flies, with the transgene or mutation of interest, are kept at 10° for 7 d. The cold temperature depolymerizes microtubules, resulting in chromosome nondisjunction during female meiosis. As soon as females are removed from the cold-shock, they are mated to C(2)EN *bw^1^,sp^1^* males in a 1:1 ratio. Of the numerous C(2)EN strains, it is important to use C(2)EN *bw^1^,sp^1^* because this strain has the greatest proportion of C(2)EN-bearing sperm (Dernburg *et al.* 1996). This is usually set-up in four bottles, each containing 75 cold-shocked virgins and 75 C(2)EN males. Only oocytes bearing either the nullo-2 or 2,2 nondisjunctional products will produce viable offspring when crossed to C(2)EN males. C(2)EN-bearing progeny are readily identified because they lack the second chromosome balancer (CyO). Alternatively, one can follow brown (*bw*) and speck (*sp*) markers on the compound chromosome. It should be noted that this approach is limited to transgenes or mutations on the X, 3rd, and 4th chromosomes. We have successfully used this method to introduce the white mutation (*w^1118^*) on the X chromosome into the C(2)EN stock (discussed in further detail in the sections to follow). We have also established C(2)EN stocks bearing the transgene, red fluorescent protein-tagged chromosome marker histone H2Av, or HisH2Av-mRFP1 [subsequently termed HisRFP (Schuh *et al.* 2007)], using an alternative method, as detailed in the sections to follow. We discuss the crosses necessary for establishing and maintaining stocks once transgenes and/or mutants have been introduced into C(2)EN-bearing flies.

Although cold-induced nondisjunction can be applied generally, this technique has some limitations. For instance, it would not work for some temperature sensitive mutations or dominant female sterile mutants. Thus, we developed an alternative method of inducing nondisjunction by using the heteroallelic combination of *bubR1* alleles {*bubR1*[*rev1*]/*bubR1*[*D1326N*] (Malmanche *et al.* 2007; Perez-Mongiovi *et al.* 2005)}. This allelic combination results in precocious sister-chromatid separation and high rates of nondisjunction during male meiosis (Malmanche *et al.* 2007). First, we constructed strains bearing the transgene HisRFP (Schuh *et al.* 2007) on the third chromosome and the aforementioned *bubR1* alleles on the second chromosome. We then crossed 80 C(2)EN *bw^1^,sp^1^*;+/+ virgin females to 60 *bubR1^rev1^/bubR1^D1326N^*;HisRFP males (Figure 3A). This cross produced large numbers of progeny bearing second chromosome markers *bw*^1^ and *sp*^1^, indicating the presence of C(2)EN. Fluorescent analysis revealed these individuals also contained the HisRFP transgene. An advantage of this method over cold-shock is that it requires fewer flies to introduce genes into the compound chromosome stocks. Disadvantages, however, are that this method is more time-consuming than the cold-shock method because the mutants and transgenes of interest must first be introduced into the *bubR1* stock. In addition, many mutants and transgenes may produce synthetic lethal phenotypes when combined with *bubR1*.

**Figure 3:**
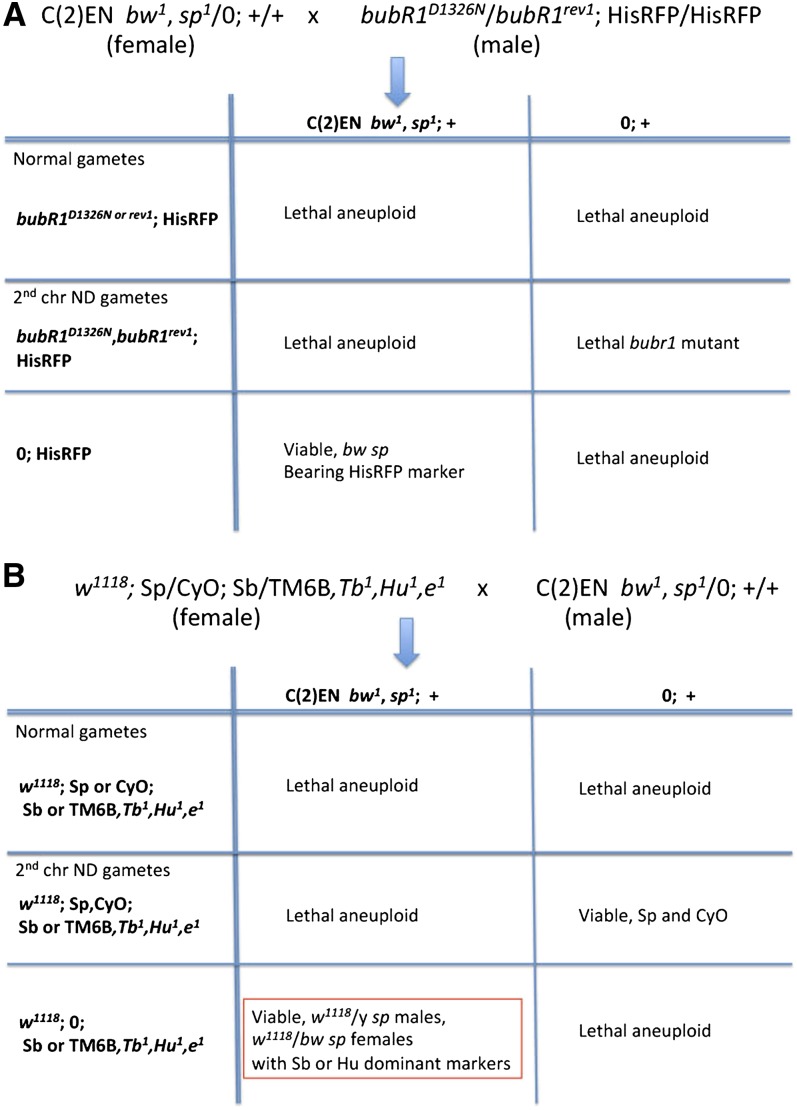
Introducing transgenes into the C(2)EN stock by promoting nondisjunction. (A) High rates of male nondisjunction were produced by a heteroallelic combination of *bubR1* mutant alleles, a spindle assembly checkpoint gene. The generation of nullo-2 gametes allows the recovery of viable C(2)EN bearing progeny. (B) High rates of female nondisjunction were produced by cold shocking virgin females. Crossing scheme for generating C(2)EN flies in a mutant white-eyed background with a third chromosome balancer. In this case, nullo-2 gametes occurred due to a cold-shock of virgin females. Constructed strains: C(2)EN *bw^1^,sp^1^*; HisRFP/+, *w^1118^*; C(2)EN *bw^1^,sp^1^*; +/+, w^1118^; C(2)EN bw^1^,sp^1^; TM6B,Tb^1^,Hu^1^,e^1^/+, w^1118^; C(2)EN bw^1^,sp^1^; HisRFP/TM6B,Tb^1^,Hu^1^,e^1^.

Although the *bubR1* mutant has proven useful for generating nondisjunction, other meiotic mutants such as *mei-s332* or *nod^DTW^* can serve a similar purpose (Kerrebrock *et al.* 1995; Wright 1974). These mutants share in common with the *bubR1* mutant, the property of high rates of chromosome nondisjunction. Therefore, one could potentially choose the mutant that causes the greatest level of chromosome nondisjunction during meiosis.

To easily identify and maintain a C(2)EN stock bearing a transgene, we took advantage of the mini-white gene associated with HisRFP. Using the cold-shock technique, we constructed a stock of C(2)EN bearing the X-linked mutant *w^1118^* and the third chromosome balancer, TM6B,*Tb^1^,Hu^1^,e^1^* (Figure 3B). These were generated by cold-shocking *w^1118^* mutant virgin flies bearing the double balancer Sp/CyO;Sb/TM6B,*Tb^1^,Hu^1^,e^1^*. After cold-shock treatment, these females were crossed to C(2)EN *bw^1^,sp^1^* males. The resultant progeny were selected for non-Sternal pleura (Sp), non-Curly wings (CyO), non-Stubble (Sb), and Humeral (Hu). Because the *w^1118^* mutation is X-linked we can easily select white-eyed males due to their X/Y genotype. We then crossed these males to their brown-eyed sisters, a marker carried by the C(2)EN flies, to generate a stable stock expressing the *w^1118^* background with the TM6B balancer. Similarly, the C(2)EN *bw^1^,sp^1^*;HisRFP/+ flies were crossed to the *w^1118^*; C(2)EN *bw^1^,sp^1^*;TM6B,*Tb^1^,Hu^1^,e^1^*/+ flies to generate and maintain a balanced stock in a white-eyed mutant background. C(2)EN white-eyed background flies in the presence of HisRFP, which also carries the mini-white gene (w^+^), show a yellow−orange eye color, thus allowing easy selection of the transgene.

These techniques allowed us to construct C(2)EN strains bearing HisRFP, thus enabling us to follow the *in vivo* dynamics of this long compound chromosome. To confirm the expected genotype, we followed chromosome segregation of third instar larval neuroblast cells from our new C(2)EN transgenic strain and control strains carrying the same fluorescent marker (Figure 4). Confirming previous results, we were able to detect the presence of the lagging C(2)EN long arms (Sullivan *et al.* 1993). We found that similar to control cells, C(2)EN cells align their chromosomes and segregate them properly to both poles (Figure 4, A and B, and Supporting Information, File S1 and File S2). Occasionally, these cells present a delay in segregating one of the chromatids but this was always resolved before cytokinesis, further confirming previous results that there are no significant errors in somatic cell mitosis (Figure 4B, white arrows).

**Figure 4:**
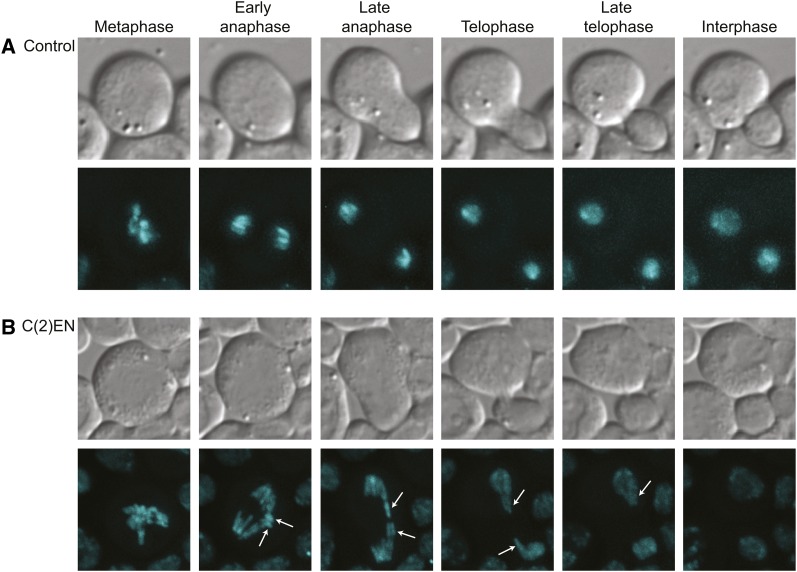
Live chromosome analysis of C(2)EN. Stills from movies of a wild-type (A) and C(2)EN-bearing (B) live larval neuroblasts progressing through mitosis. The long arms of the compound chromosome are readily observed lagging during anaphase (white arrows). Chromosomes are labeled with HisRFP (cyan), and DIC images are shown in gray.

In summary, we developed two complementary methods to introduce mutations or transgenes into the C(2)EN stock. If the desired mutation is cold sensitive or important for female meiosis, then the approach should be to promote male nondisjunction using the *bubR1* alleles. On the other hand, if the mutation has the potential to produce a genetic interaction with the spindle assembly checkpoint, it may result in lethality, and the cold induced nondisjunction approach should be chosen. Here we have focused on introducing mutant alleles and transgenes in C(2)EN but these techniques can be readily applied to other compound stocks as well. For example, the cold-shock technique can be used to create nondisjunction of the third chromosome to generate a C(3)EN stock bearing a mutant or transgene on the X, 2nd, or 4th chromosome.

## Supplementary Material

Supporting Information
