# Supplementary material for: Strategies for Outcrossing and Genetic Manipulation of Drosophila Compound Autosome Stocks
Source: G3 (Bethesda). 2013 Jan 1;3(1):1–4. doi: 10.1534/g3.112.004481 (PMC3538334; doi:10.1534/g3.112.004481)
Supplement: Supporting Information [file supp_3_1_1__index.html]

Supporting Information 

# Strategies for Outcrossing and Genetic Manipulation of Drosophila Compound Autosome Stocks

## Supporting Information for Martins *et al.*, 2013

**Files in this Data Supplement:**

- File S1 - Live analysis of HisRFP in a mitotic control larval neuroblast cell (.avi, 3.5 MB)
- File S2 - Live analysis of HisRFP in a mitotic C(2)EN larval neuroblast cell (.avi, 8.7 MB)
